# Endophytic *Penicillium* species secretes mycophenolic acid that inhibits the growth of phytopathogenic fungi

**DOI:** 10.1111/1751-7915.14203

**Published:** 2023-01-26

**Authors:** Neri Azar, Orna Liarzi, Maor Zavitan, Mohamed Samara, Ahmed Nasser, David Ezra

**Affiliations:** ^1^ Department of Plant Pathology and Weed Research ARO – the Volcani Center Rishon LeZion Israel; ^2^ Institute of Soils, Water and Environmental Sciences ARO – the Volcani Center Rishon LeZion Israel

## Abstract

The worldwide demand for reduced and restricted use of pesticides in agriculture due to serious environmental effects, health risks and the development of pathogen resistance calls for the discovery of new bioactive compounds. In the medical field, antibiotic‐resistant microorganisms have become a major threat to man, increasing mortality. Endophytes are endosymbiotic microorganisms that inhabit plant tissues without causing any visible damage to their host. Many endophytes secrete secondary metabolites with biological activity against a broad range of pathogens, making them potential candidates for novel drugs and alternative pesticides of natural origin. We isolated endophytes from wild plants in Israel, focusing on endophytes that secrete secondary metabolites with biological activity. We isolated 302 different endophytes from 30 different wild plants; 70 of them exhibited biological activity against phytopathogens. One biologically active fungal endophyte from the genus *Penicillium*, isolated from a squill (*Urginea maritima*) leaf, was further examined. Chloroform‐based extraction of its growth medium was similarly active against phytopathogens. High‐performance liquid chromatography separation followed by gas chromatography/mass spectrometry analysis revealed a single compound—mycophenolic acid—as the main contributor to the biological activity of the organic extract.

## Graphical abstract

An endophytic Penicillium sp. was isolated from a leaf of a squill (*Urginea maritima*). Both the endophyte and the chloroform extract of its growth medium inhibited the growth of phytopathogenic fungi in vitro. HPLC separation revealed a single peak, which was similarly active to the crude organic extract. GC/MS analysis of this peak suggested that the active compound is mycophenolic acid.

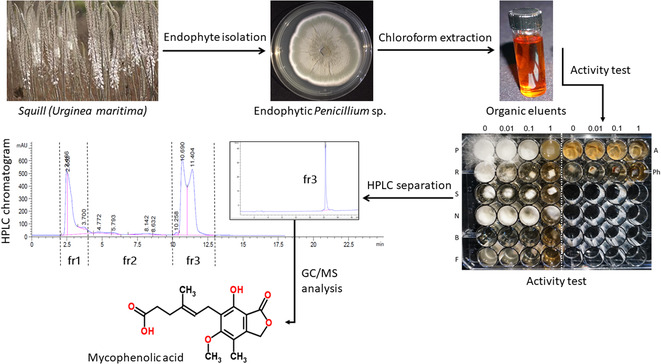

## INTRODUCTION

Endophytes are microorganisms that reside in the living internal tissues of the plant without showing any apparent symptoms of their presence (Patil & Maheshwari, 2021). These microorganisms include both fungi and bacteria (Ek‐Ramos et al., 2019; Porras‐Alfaro & Bayman, 2011). Endophytic microorganisms are found in virtually every plant on earth (Hardoim et al., 2015; Strobel et al., 2004) and can be isolated from all plant compartments (Ryan et al., 2008). They confer fitness benefits to their hosts, such as growth enhancement, increased reproductive success and tolerance to biotic and abiotic stresses (Liarzi & Ezra, 2014). Moreover, the beneficial effects of endophytes are not limited to their hosts; they can also be obtained by introducing endophytes, isolated from wild relatives of cultivated crops, as bioinoculants in their close cultivars (Mili et al., 2021).

Microbial secondary metabolites are low‐molecular mass products of secondary metabolism, which are not essential for the growth of the producing culture, but serve diverse survival functions in nature (Sanchez & Demain, 2011), acting as agents that help endophytic fungi compete and survive (Alam et al., 2021). In the past few years, hundreds of new metabolites, originating from plant endophytic fungi, have been characterized (Zheng et al., 2021). These bioactive metabolites can serve as candidates for novel drugs (see reviews by Patil et al., 2016, Lee & Shim, 2020, Manganyi & Ateba, 2020, Tiwari & Bae, 2022) and as pesticides of natural origin (Xu et al., 2021).

The genus *Penicillium* is ubiquitous; it can be isolated from diverse extreme environments as well as from plants (epiphytic, endophytic and rhizospheric) and decaying fruit (Yadav et al., 2018). Many *Penicillium* species produce a vast array of biologically active secondary metabolites, making them good candidates for agricultural, biotechnological and pharmaceutical applications (Deshmukh et al., 2022; Jouda et al., 2016; Koul et al., 2016; Kumari et al., 2021; Toghueo & Boyom, 2020; Wen et al., 2022; Xie et al., 2018). In light of the endophytes' beneficial effects on their host plant, together with their ability to produce biologically active secondary metabolites, we screened endophytes isolated from wild plants in Israel for producers and secretors of secondary metabolites that are active against phytopathogenic fungi and bacteria. One endophyte, from the genus *Penicillium*, was further characterized, and its active compound—mycophenolic acid—was identified.

## EXPERIMENTAL PROCEDURES

### Plant sources

A broad range of native plants was surveyed for the presence of potential fungal and bacterial endophytes. All plants were collected from their natural habitats in Israel during the different seasons. At least one tissue type was sampled from each plant (Table S1). The fresh samples were put in paper bags and taken to the laboratory under cooling conditions. Plants were identified using the Flora of Israel online database (https://flora.org.il/en/plants/). Seeds that had been collected in their natural habitats were purchased from the commercial nursery ‘Seeds from Zion’ (Kerem Maharal, Israel).

### Endophyte isolation

The plant stems/branches, leaves, flowers, roots, bulbs/tubers and fruit were washed with running tap water. Next, the plant parts were surface‐sterilized by immersing in a 2% sodium hypochlorite solution for 1 min (leaves, flowers, fruit and seedlings) or 2 min (stems, branches, bulbs and tubers). Then, the samples were rinsed twice with double‐distilled water for 5 min each time. The surface‐sterilized plant samples were cut into small segments and placed on nutrient agar (NA) (Acumedia) for bacterial endophytes, and 25% potato dextrose agar (PDA) (Acumedia) amended with tetracycline at 12 μg/mL (Sigma) to isolate fungal endophytes. Each media plate contained five pieces of plant tissue. All plates were incubated at 25°C, and checked daily for 10 days for any fungal or bacterial growth. Single fresh bacterial and fungal colonies were transferred, using a 1‐mm bacterial needle, to NA and 100% PDA media plates, respectively, and further incubated at 25°C. All endophytes that originated from the same plant and presented similar phenotypical characteristics were pooled together and one representative endophyte was selected for activity tests.

Endophyte isolation from seeds was carried out as described above, except that after surface sterilization, the seeds were ground into small pieces using a mortar and pestle, which were then placed on the appropriate media. Alternatively, the surface‐sterilized seeds were put on agar–water plates (Romical) to induce germination, and then, the germinated seedlings were surface‐sterilized by immersing in 2% sodium hypochlorite solution for 1 min, cut into small pieces and put on media plates for endophyte isolation.

### Bioactivity screening

The ability of the endophytic isolates to inhibit phytopathogenic fungi was examined against three different phyla: Ascomycota—*Alternaria alternata*, *Botrytis cinerea*, *Fusarium oxysporum*, *Phoma tracheiphila*, *Sclerotinia sclerotiorum*, *Neoscytalidium dimidiatum*; Basidiomycota—*Rhizoctonia solani*; and Oomycota—*Pythium ultimum*. The biological activity of endophytic isolates was also examined against Gram‐negative bacteria—*Erwinia amylovora*, *Escherichia coli*, *Xanthomonas* sp.; and against Gram‐positive bacteria—*Clavibacter michiganensis* subsp*. michiganensis*. All cultures were maintained routinely on PDA or NA media plates for fungi and bacteria, respectively, at 25°C, and renewed every 1–2 weeks.

The antibiosis tests were performed using 100% PDA, 100% NA and 50% PDA + 50% NA media plates for fungi, bacteria or a combined test for fungi and bacteria, respectively. Each endophyte was tested once, in duplicates, against all of the listed fungi and bacteria. For fungal endophytes, a fresh fungal mycelium plug was transferred to the middle of the suitable growth media plate and the fungus was grown to a diameter of 15–20 mm (approximately 3–4 days at 25°C). For bacterial endophytes, a smear from a fresh bacterial culture plate was drawn in a straight line, the letter ‘Y', or the letter ‘X', which divided the test plate into two, three or four sections, respectively, and the culture plates allowed to grow for 2–3 days at 25°C. Next, a plug of PDA harbouring mycelia of the test fungus or oomycete, or a smear from a test bacteria culture, was placed in the test plate, without any direct contact with the endophyte already grown in the plate. In parallel, as a control, each test organism was grown under the same conditions in the absence of the endophyte. The control and test plates were incubated at 25°C, and examined after 1, 3 and 7 days. The activity of the endophyte Uml2 was determined after 3 days incubation of the culture plates at 25°C. Active endophyte was defined as inducing at least 30% inhibition of the growth of the test organism. For phytopathogenic fungi, this meant at least 30% reduction in their colony diameter in the presence of the endophyte relative to the growth measured in its absence, whereas for bacteria, it meant a reduction in bacterial growth, determined by visual assessment of the colony's density.

### Molecular identification of fungal endophyte isolate UMl2

A single‐spore colony of endophyte UMl2 was prepared. DNA was extracted using the Quick‐DNA Fungal/Bacterial Miniprep Kit (Zymo Research) according to the manufacturer's instructions. Molecular identification was performed using amplification of the internal transcribed spacer 5.8 S rDNA (ITS), *β‐tubulin*, *calmodulin* and *RNA polymerase II* (*RPB2*). The ITS region was amplified using primers ITS1 (TCCGTAGGTGAACCTGCGG) and ITS4 (TCCTCCGCTTATTGATATGC), *β‐tubulin* using primers Bt2a (GGTAACCAAATCGGTGCTGCTTTC) and Bt2b (ACCCTCAGTGTAGTGACCCTTGGC) (Glass & Donaldson, 1995), *calmodulin* using primers Cmd5 (CCGAGTACAAGGAGGCCTTC) and Cmd6 (CCGATAGAGGTCATAACGTGG) (Hong et al., 2006) and *RPB2* using primers 5F (GAYGAYMGWGATCAYTTYGG) and 7cR (CCCATRGCTTGYTTRCCCAT) (Liu et al., 1999). All amplifications were performed in a 25‐μL reaction mix containing 1 μl (10 ng/μl) DNA, 1 μl (10 μM) of each primer, 9.5 μl ddH_2_O (Fisher Scientific) and 12.5 μl Dream Mix Taq (Thermo Scientific), using a Labcycler (SensoQuest GmbH).

The PCR programme for ITS was as follows: denaturation at 96°C for 2 min; 40 cycles of 96°C for 45 s, 55°C for 45 s and 72°C for 1 min, 5 min at 72°C; for *β‐tubulin*: denaturation at 95°C for 5 min; 32 cycles of 95°C for 1 min, 55°C for 1 min and 72°C for 1 min, 5 min at 72°C; for *calmodulin*: denaturation at 94°C for 5 min; 35 cycles of 94°C for 45 s, 55°C for 45 s and 72°C for 1 min, 7 min at 72°C; for *RPB2*, TOUCH‐UP amplification was performed as follows: denaturation at 94°C for 5 min; 5 cycles of 94°C for 45 s, 50°C for 45 s and 72°C for 1 min; an additional 5 cycles of 94°C for 45 s, 52°C for 45 s and 72°C for 1 min; and 30 cycles of 94°C for 45 s, 55°C for 45 s and 72°C for 1 min, 7 min at 72°C.

PCR products were examined by electrophoresis in a 1.2% agarose gel (Sambrook & Russell, 2001) and were purified using a PCR purification kit (MEGAquick‐spin, iNtRON, South Korea) according to the manufacturer's instructions. Purified products were sent for direct PCR sequencing by Macrogen. The sequences obtained in the present study were compared with those already present in the GenBank database by applying BLAST software on the National Center for Biotechnology Information website (http://www.ncbi.nlm.nih.gov/BLAST/). Identity of at least 95% was set for genus identification.

### Chloroformic extraction of UMI2 growth medium

Three plugs of growing mycelium of the endophyte UMl2 were placed in an Erlenmeyer flask (0.5 L) containing 0.25 L potato dextrose broth (PDB) (Acumedia), and were grown at 25°C for 10–14 days. The organic extraction was performed as follows: The growing medium was filtered through gauze sheets into a clean beaker. An equal volume of the organic solvent chloroform (Bio Lab, Jerusalem, Israel) was added, and the mixture was stirred by magnetic stirrer at increasing velocity until the emulsion was homogeneous. The emulsion was then transferred to a 2‐L separatory funnel, mixed vigorously and left to stand until phase separation. Each phase was collected and stored separately. Organic extraction of the growth medium phase was repeated three times sequentially, with fresh chloroform each time. The organic phase was transferred to a 1‐L glass vacuum flask and evaporated using Rotavapor‐R (BÜCHI Labortechnik AG) with vacuum pump (KNF LABOPORT) and a water temperature of 40°C. The dried organic extraction was dissolved in fresh chloroform and transferred using a glass pipette into a 9‐mL glass vial (S Murray & Co). Then, the organic extract was dried again by chloroform evaporation using a flow of dry nitrogen gas together with sample heating to 40°C. The weight of the organic extract was calculated as the difference in vial weight before and after the addition of the organic extract. Finally, the organic extract was dissolved in acetonitrile (Bio Lab) to a concentration of 10 mg/ml for further analyses.

### Activity tests for the chloroform extract

The activity tests were performed in a sterile 48‐well plate (Cellstar, Greiner Bio‐One). Each of the eight phytopathogenic fungi listed above was exposed to three increasing concentrations of the crude organic extract: 0.01 mg/ml, 0.1 mg/ml and 1 mg/ml. As a control, each fungus was exposed to a similar volume (0.1 ml) of pure acetonitrile. After complete evaporation of the organic solvent from the plate, 1 ml PDB was added to each well and a plug (0.16 mm^2^) of growing mycelium of the test fungus was inserted. Fungal growth was assessed visually after 3 days incubation at 25°C, except for the slow‐growing fungus *P. tracheiphila*, which was assessed after 7 days of incubation.

### HPLC separation of the chloroform extract

Chloroform extract analysis and separation were carried out using both analytical ultra‐high‐performance liquid chromatography (UHPLC) (Infinity 1290) and preparative HPLC (Infinity 1260) (Agilent Technologies). First, the chloroform extract (0.2 mg/mL) was filtered through a 0.22‐μm Teflon filter (Starlab Scientific, Scientific Instruments Ltd.) and loaded into the analytical UHPLC to calibrate the optimal conditions for separation. The column was a Luna C18 (100 A, 250 × 4.6 mm, 5 μm) (Phenomenex). The flow rate was 0.8 ml/min, column oven temperature was 30°C, injection volume was 5 μl and the absorption wavelength was 280 nm. All solvents were HPLC grade, and the water was supplemented with 0.1% acetic acid (Bio Lab). The initial mobile phase was composed of 30% water (solvent A) and 70% acetonitrile (solvent B) run in a gradient to 90% solvent B over 20 min. Next, the chloroform extract was subjected to higher scale separation using preparative HPLC under the following conditions: Kinetex EVO C18 column (5 μm, 100 A, 250 × 21.2 mm) (Phenomenex); flow rate 20 ml/min, column oven temperature 25°C, injection volume 10 ml and absorption wavelengths 220, 240 and 280 nm. The solvents were as indicated for the UHPLC. Separation (18 min) was carried out using a mixture of 70% solvent A and 30% solvent B run in gradient as follows: 0–10 min to 42% solvent B, 10–16 min to 44% solvent B, 16–18 min back to initial conditions. The different fractions obtained in the preparative HPLC were dried in a rotary evaporator and used for activity tests as described for the chloroform extract. Last, each fraction was analysed using the analytical UHPLC, under the same conditions indicated above except that the initial ratio was 80% solvent A and 20% solvent B, followed by an increase in solvent B to 80% within 15 min, and then a return to initial conditions over 2 min.

### Mycophenolic acid identification

The single compound of active fraction 3 from the HPLC separation was identified by gas chromatography/mass spectrometry (GC/MS) analysis. First, the compound was supplemented with a trialkylsilyl group by the addition of 0.2 ml bis(trimethylsilyl)trifluoroacetamide (BSTFA, derivatization‐grade, Sigma Aldrich), and incubation for 2 h at 70°C. Analysis was performed with a GC 7890B, MSD 5977A, GC Sampler 80 (Agilent), on an HP‐5MS 5% phenyl methyl silox column (1.33 m × 150 μm × 0.25 μm Agilent). The separation conditions were injector temperature 240°C, split injection (ratio 1:10) and injection volume 2 μl. Recorded mass range was 40–800 m/z, separation time 29.5 min. Oven temperature was 40°C increased to 280°C at a rate of 15°C/min, and held for 8 min. Then, the temperature was increased at a rate of 10°C/min to a final temperature of 300°C, and held for an additional 2 min. The GC/MS spectrum profiles were analysed with MassHunter software (Agilent). The volatiles were identified by comparison of their retention indices with published values and with spectral data obtained from NIST Mass Spectral Library, ver. 2.2, 2014. The suggested name of the compound was obtained using the ChEMBL database (https://www.ebi.ac.uk/chembl/).

Identification of the single compound of fraction 3 was confirmed by comparing both its analytical and activity characteristics to those of commercially available mycophenolic acid (Fermentek Ltd). The commercial mycophenolic acid (100 ppm) was silylated, injected into the GC/MS and analysed as described above. The mycophenolic acid was also co‐injected with fraction 3 into the analytical UHPLC. The separation conditions were as described for fraction 3, except that the column was a Luna Omega 3 μm SUGAR (100 A, 150 × 4.6 mm; Phenomenex). The activity tests were performed as described above for the chloroform extract, except that the commercial mycophenolic acid was dissolved in methanol (Bio Lab) to a concentration of 10 mg/mL.

## RESULTS

### Collection of endophytes

The sources for the 146 bacterial and 156 fungal endophytes isolated in this study were 30 wild plants from seven geographical areas in Israel (Table 1). Plant seeds and bulbs/tubers presented the highest ratio of endophyte isolation, with bacterial endophytes more abundant in the former and fungal endophytes more abundant in the latter. In contrast, fruit, leaves and stems/branches gave the lowest ratio of endophyte isolation (Table S1). Fungal endophytes were more abundant than bacterial endophytes in most plant parts except for seeds and germinated seeds, in which the bacterial endophytes were more abundant, and flowers, in which their ratio was equal (1.8 endophytes/plant part, Table 1).

**TABLE 1:** Total number of endophytes and active endophytes in different plant parts.

		Isolated endophytes		Ratio endophyte/plant part			Active endophytes		Ratio active endophyte/plant part		
Plant part	Collected plant tissues (N)	Bacteria (N)	Fungi (N)	Bacteria	Fungi	Total	Bacteria (N)	Fungi (N)	Bacteria	Fungi	Total
Stem/branch	23	26	38	1.13	1.65	2.78	8	9	0.35	0.39	0.74
Leaf	21	26	33	1.24	1.57	2.81	1	5	0.05	0.24	0.29
Flower	10	18	18	1.80	1.80	3.60	4	4	0.40	0.40	0.80
Root	10	18	26	1.80	2.60	4.40	2	12	0.20	1.20	1.40
Fruit	8	8	13	1.00	1.63	2.63	4	3	0.50	0.38	0.88
Seed	8	41	14	5.13	1.75	6.88	10	1	1.25	0.13	1.38
Bulb/tuber	3	8	12	2.67	4.00	6.67	2	5	0.67	1.67	2.33
Germinated seed	1	2	1	2.00	1.00	3.00	0	0	0.00	0.00	0.00

### Endophyte screening for biological activity

Among the 103 bacterial and 103 fungal endophytes tested, only 31 and 39, respectively, exhibited >30% biological activity against phytopathogenic bacteria and fungi (Table 1). The highest ratio of active endophytes per plant part was for bulbs/tubers, which also displayed the highest ratio for active fungal endophytes (Table 1). The highest ratio of active bacterial endophytes was in the seeds. The lowest ratio of active bacterial and fungal endophytes was in the leaves and seeds, respectively, and endophytes isolated from germinated seeds exhibited no biological activity. Interestingly, the ratio of active bacterial endophytes per flower was equal to that of active fungal endophytes (0.4 active endophytes/flower; Table 1), in accordance with the equal ratios obtained for total number of bacterial and fungal endophytes isolated from flowers. In contrast, the largest difference between the ratio of active bacterial and fungal endophytes per tissue was obtained in roots (sixfold more fungal than bacterial) and seeds (13‐fold more bacterial than fungal) (Table 1).

### Fungal endophyte isolate UMl2

One fungal endophyte, UMI2, isolated from a squill (*Urginea maritima*) leaf collected from the Judaean mountains, exhibited 30–70% growth inhibition of *S. sclerotiorum* and *P. tracheiphila*, and 0–30% growth inhibition of *A. alternata*, *B. cinerea*, *R. solani* and *F. oxysporum*, in comparison to the fungal growth in the absence of the endophyte, in dual‐culture activity assays. This endophyte did not inhibit the growth of *N. dimidiatum* or *P. ultimum*. Molecular identification based on ITS, *β‐tubulin*, *calmodulin* and *RPB2* sequences with 94%, 93%, 99% and 86% coverage, respectively, revealed that the fungus belongs to the genus *Penicillium* (99.5%, 99.8%, 99.6% and 96.5% identity, respectively), probably *P. momoii* (all four sequences) or *P. rubefaciens* (all sequences except *RPB2*). All sequences were deposited in GenBank (NCBI) as ITS—ON407109, *β‐tubulin*—ON420223, *calmodulin*—ON420224 and *RPB2*—ON492034.

### Activity tests with the chloroform extract of UMI2 growth medium

**FIGURE 1:**
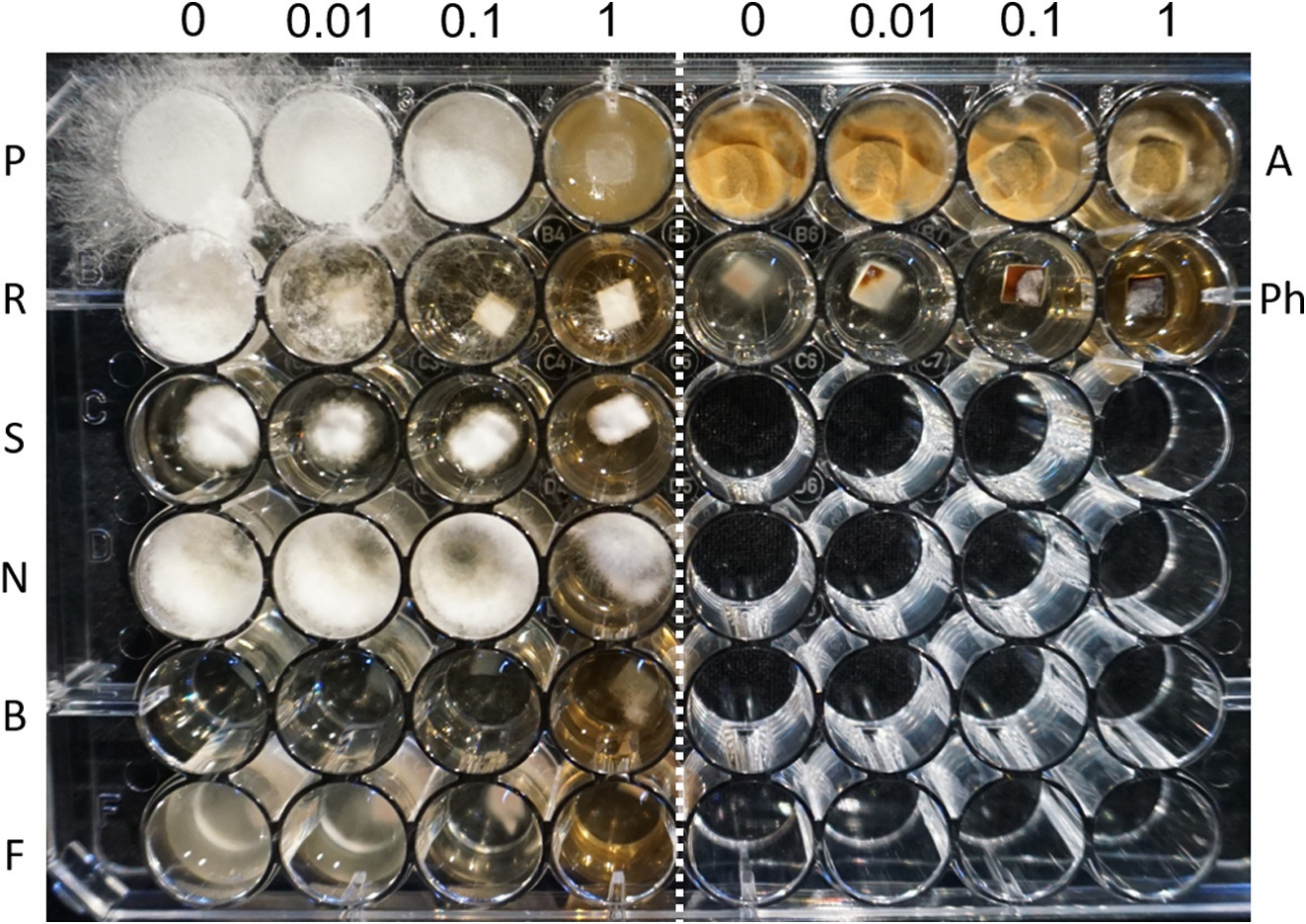
Activity tests with crude organic extract of endophyte UMl2. Increasing concentrations of the crude organic extract (numbers above the ELISA plate, mg/mL) were placed in each well. After organic solvent evaporation, 1 ml of PDB was added to each well and a PDA plug harbouring the tested fungus was transferred. The phytopathogenic fungi were: P—*P. ultimum*, R—*R. solani*, S—*S. sclerotiorum*, N—*N. dimidiatum*, B—*B. cinerea*, F—*F. oxysporum*, A—*A. alternata* and Ph—*P. tracheiphila*. The plates were incubated at 25°C for 3 days and the minimal concentration of the crude extract that inhibited fungal growth was determined.

To further understand the basis of the bioactivity of endophyte UMI2, we extracted the organic eluents secreted by the fungus during its growth using chloroform. As shown in Table 2 and Figure 1, the crude organic extract inhibited the growth of the tested phytopathogenic fungi to various degrees. The most sensitive fungi were *R. solani*, *S. sclerotiorum* and *P. tracheiphila*, with minimal concentration for inhibition of 0.01 mg/ml. These were followed by *P. ultimum* and *F. oxysporum*, with minimal concentration for inhibition of 0.1 mg/ml. The least sensitive fungi were *N. dimidiatum* and *A. alternata*, with minimal concentration for inhibition of 1 mg/ml, whereas the crude organic extract did not inhibit the growth of *B. cinerea*. These results suggest that the organic eluents of the endophyte UMl2 possess biological activity against various phytopathogenic fungi.

**TABLE 2:** Minimal concentration inhibiting each phytopathogenic fungus.

		HPLC separation			Mycophenolic acid (mg/ml)
Tested fungus	Organic extract (mg/ml)	Fraction 1 (mg/ml)	Fraction 2 (mg/ml)	Fraction 3 (mg/ml)	
*P. ultimum*	0.1^a^	1	1	0.1	0.1
*R. solani*	0.01	1	1	0.01	0.01
*S. sclerotiorum*	0.01	1	0.1	0.01	0.01
*N. dimidiatum*	1	‐^b^	‐	1	‐
*A. alternata*	1	‐	‐	1	‐
*F. oxysporum*	0.1	‐	1	0.1	0.1
*P. tracheiphila* ^c^	0.01	1	0.1	0.01	0.01

a The minimal concentration (mg/mL) that inhibits the tested fungus after 3 days exposure.

b No inhibition obtained at the highest concentration (1 mg/ml) tested.

c The results for *P. tracheiphila* were obtained after 7 days exposure due to the slow growth of this fungus.

### Mycophenolic acid is the active compound

**FIGURE 2:**
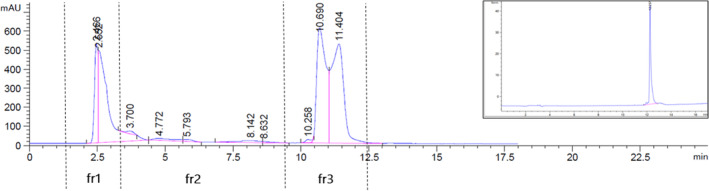
HPLC separation chromatogram of crude organic extract of endophyte UMl2. Chloroform extract of UMl2 eluent was separated in a reverse phase C18 column. Three fractions, designated fr1, fr2 and fr3 with retention times of 2–4 min, 4–10 min and 10–13 min, respectively, were collected. Insert, fr3 after further purification.

**FIGURE 3:**
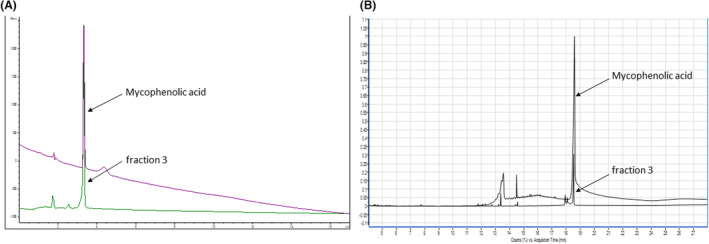
Comparison of fraction 3 from the HPLC separation of UMl2 eluent and mycophenolic acid. Fraction 3 and mycophenolic acid (100 ppm) were co‐injected for UHPLC (A) and GC/MS (B) analyses. The retention times of both eluents were identical: 3 and 18.5 min for UHPLC and GC/MS respectively.

To further elucidate which compound in the organic extract of the endophyte UMl2 is responsible for its biological activity, we used HPLC to segregate different compounds in the crude extract. We collected three fractions based on the ‘peak’ (representing the absorption of a compound) patterns on the HPLC chromatogram and their retention times: 2–4 min, 4–10 min and 10–13 min, designated as fractions 1, 2 and 3 respectively (Figure 2). Each fraction contained several compounds, represented by several peaks in the chromatogram; however, further separation of fraction 3 resulted in a single peak with retention time of 12.26 min (Figure 2, insert). We performed activity tests with each fraction to determine which is more potent against the tested phytopathogenic fungi. As shown in Table 2, all three fractions were biologically active, but fraction 3 was the most active—it inhibited the growth of the tested fungi at lower concentrations. GC/MS analysis of the purified fraction 3 revealed, with 97.1% probability, a single compound with empirical formula C_17_H_20_O_6_, and molecular weight of 320.34 g/mol. Based on the ChEMBL database, we suggest that this compound is mycophenolic acid. We validated this suggestion both analytically (Figure 3) and biologically (Table 2). As shown in Figure 3, the retention time of the purified fraction 3 was identical to that of commercial mycophenolic acid on both UHPLC (3 min, Figure 3A) and GC/MS (18.5 min, Figure 3B) chromatograms. As shown in Table 2, the ability of commercial mycophenolic acid to inhibit the growth of the tested fungi was identical to that of fraction 3, except for the fungi *N. dimidiatum* and *A. alternata*, which were slightly inhibited by fraction 3 (minimal concentration of inhibition of 1 mg/ml), but not by mycophenolic acid. Since mycophenolic acid is the main compound of fraction 3 (Figure 3) and the latter is the most active fraction in the organic extract of UMl2 eluate (Table 2), we suggest that mycophenolic acid is the main contributor to endophyte UMl2's ability to inhibit phytopathogenic fungi.

## DISCUSSION

There is a pressing need to search for novel, affordable and nontoxic natural bioactive compounds with potential application in medicine and agriculture. Endophytic fungi are promising candidates based on their ability to produce diverse biologically active secondary metabolites (Adeleke & Babalola, 2021; Tiwari & Bae, 2022; Wen et al., 2022; Xu et al., 2021; Zheng et al., 2021). There are various tools for screening potentially bioactive fungal endophytes, depending on their sporulation and cultivation properties (Sun & Gou, 2012; Vasundhara et al., 2016). In the current study, all 302 fungal and bacterial endophytes were culturable and could be isolated from different plant parts (Table S1).

Transmission of fungal and bacterial endophytes can occur either vertically via seeds or horizontally through the environment (Frank et al., 2017; Shahzad et al., 2018). Accordingly, seeds exhibited the highest ratio of endophytes per plant part, with bacterial endophytes being more abundant than fungal endophytes (Table 1). In addition, underground parts (bulb/tuber and root) demonstrated a higher ratio of endophytes (both total count and active) per plant part than aboveground parts (stem/branch, leaf, flower, fruit and germinated seeds), with the exception of seeds. Differences in endophyte diversity between above‐ and underground plant parts have been shown elsewhere (Dong et al., 2018; Kandel et al., 2017).

The fungal endophyte isolated from a squill leaf, of the genus *Penicillium*, inhibited the growth of various phytopathogenic fungi belonging to the phyla Basidiomycota and Ascomycota, but not Oomycota. Since the antibiosis tests were performed in vitro, this suggests that the endophytic *Penicillium* secretes bioactive metabolites into the growth media that affect the growth of the phytopathogenic fungi. The production of active metabolites by endophytic *Penicillium* is well known (Deshmukh et al., 2022; Jouda et al., 2016; Koul et al., 2016; Kumari et al., 2021; Toghueo & Boyom, 2020; Wen et al., 2022; Xie et al., 2018). Both the fungal endophyte and its eluents extracted using the organic solvent chloroform strongly inhibited *S. sclerotiorum* and *P. tracheiphila*. However, there were differences in inhibitory activity between the whole organism and its crude organic extract. For example, only the former managed to inhibit *B. cinerea*, whereas only the latter inhibited *P. ultimum* and *N. dimidiatum*. A possible explanation is that another compound(s) secreted by the endophytic fungus, but not extracted using chloroform, is needed to inhibit *B. cinerea*. Alternatively, the concentration of the compounds in the crude organic extract might be higher than when they are secreted by the fungus during the antibiosis tests, thereby enabling inhibition of the phytopathogenic fungi *P. ultimum* and *N. dimidiatum*. Differences in biological activity between the fungus and its secreted metabolites have been demonstrated elsewhere (Charria‐Girón et al., 2021; Liarzi et al., 2016).

HPLC and GC/MS analyses demonstrated that a single compound, mycophenolic acid, is the main contributor to the biological activity of the crude organic extract (Figure 3, Table 2). Mycophenolic acid is a fungal metabolite with an acetate‐mevalonate ring system and a terpenoid tail (Stierle & Stierle, 2015). Numerous *Penicillium* species produce mycophenolic acid (Muth & Nash III, 1975; Vinokurova et al., 2005), and in the current study, we found that *Penicillium momoii* or *Penicillium rubefaciens*, both of which belong to the *Penicillium corylophilum* phylogenetic clade (Visagie et al., 2016), produce and secrete this compound. Mycophenolic acid has a broad spectrum of activity: antifungal, antibacterial, antiviral, antitumour, immunosuppressive and antipsoriatic (see reviews by Kitchin et al., 1997, Stierle & Stierle, 2015 and references therein). Accordingly, our results showed antifungal activity of mycophenolic acid (Table 2). Similar antifungal activity has been demonstrated previously (Anderson et al., 1988; Florey et al., 1946).

Although mycophenolic acid was the most active compound produced by endophytic *Penicillium* isolated from *Urginea maritima*, and despite its antifungal activity, this compound's application in agriculture is unlikely, due to its negative side effects (Kitchin et al., 1997). Thus, further examination of the other active endophytes isolated in this study (Table 1) and their eluents is needed to find additional biologically active compounds that would be potential candidates to control phytopathogenic fungi in agriculture. Alternatively, these active endophytes might be candidates towards sustainable agriculture, as biocontrol agents alone or as a component of integrated pest management; however, advance investigations regarding the effects of the endophyte on the host plant as well as the development of successful endophyte application technologies are required (Tripathi et al., 2022; Verma et al., 2022).

## AUTHOR CONTRIBUTIONS

**Neri Azar:** Conceptualization (equal); data curation (lead); formal analysis (lead); investigation (lead); writing – review and editing (equal). **Orna Liarzi:** Conceptualization (equal); formal analysis (equal); writing – original draft (equal); writing – review and editing (equal). **Maor Zavitan:** Conceptualization (equal); data curation (equal); investigation (equal). **Mohamed Samara:** Data curation (supporting); formal analysis (supporting); writing – review and editing (equal). **Ahmed Nasser:** Data curation (supporting); formal analysis (supporting); writing – review and editing (supporting). **David Ezra:** Conceptualization (equal); formal analysis (equal); project administration (lead); supervision (lead); writing – original draft (equal); writing – review and editing (equal).

## CONFLICT OF INTEREST

The authors have no conflict of interest to declare.

## Supporting information


Table S1.
