# Supplementary material for: Endophytic Penicillium species secretes mycophenolic acid that inhibits the growth of phytopathogenic fungi
Source: Microb Biotechnol. 2023 Jan 26;16(8):1629–38. doi: 10.1111/1751-7915.14203 (PMC10364310; doi:10.1111/1751-7915.14203)
Supplement: Supplementary file 1 — Table S1. [file MBT2-16-1629-s001.docx]

**Table 1.** Source of endophytes.

|  | | Tissue type | | | | | | | | Isolated endophytes | |
| --- | --- | --- | --- | --- | --- | --- | --- | --- | --- | --- | --- |
| Plant species | Geographical location | Stem/branch | Leaf | Flower | Root | Bulb/tuber | Seed | Germinated seed | Fruit | Bacteria (N) | Fungi (N) |
| *Calotropis procera* | Dead sea | + | + |  |  |  | + |  | + | 6 | 0 |
| *Ziziphus spina-christi* | Coastal plain | + | + |  |  |  |  |  |  | 0 | 5 |
| *Oenothera drummondii* | Coastal plain | + | + | + |  |  |  |  |  | 4 | 4 |
| *Capparis sinaica* | Arava desert | + |  |  |  |  |  |  |  | 2 | 0 |
| *Ephedra foeminea* | Arava desert | + |  | + |  |  | + | + | + | 8 | 11 |
| *Ricinus communis* | Judaean foothills | + | + | + |  |  | + |  |  | 3 | 1 |
| *Arbutus andrachne* | Judaean mountains | + | + |  |  |  |  |  |  | 1 | 3 |
| *Rubus sanguineus* | Judaean mountains | + |  |  |  |  |  |  |  | 2 | 0 |
| *Ficus carica* | Judaean mountains | + |  |  |  |  |  |  |  | 1 | 2 |
| *Olea europae* | Judaean mountains | + | + |  |  |  |  |  |  | 1 | 3 |
| *Sarcopoterium spinosum* | Judaean mountains | + | + |  | + |  | + |  |  | 12 | 9 |
| *Pistacia lentiscus* | Judaean mountains | + | + |  |  |  |  |  | + | 4 | 5 |
| *Arum palaestinum* | Judaean mountains | + | + |  | + |  |  |  |  | 6 | 4 |
| *Urginea maritima* | Judaean mountains |  | + |  | + | + |  |  |  | 7 | 11 |
| *Cyclamen persicum* | Judaean mountains | + | + | + | + | + |  |  |  | 10 | 18 |
| *Allium neapolitanum* | Mount Carmel | + | + |  | + | + |  |  |  | 8 | 10 |
| *Ruta chalepensis* | Mount Carmel | + | + |  | + |  |  |  |  | 8 | 10 |
| *Daucus bicolor* | Mount Carmel | + | + | + | + |  |  |  |  | 4 | 9 |
| *Calicotome villosa* | Mount Carmel | + | + | + |  |  |  |  |  | 3 | 6 |
| *Rough bindweed* | Mount Carmel |  | + |  |  |  |  |  | + | 3 | 5 |
| *Narbonne Star-of-Bethlehem* | Mount Carmel | + |  |  | + |  |  |  | + | 7 | 6 |
| *Nerium oleander* | Hashofet stream | + | + | + |  |  |  |  |  | 9 | 6 |
| *Alcea setosa* | Hashofet stream | + | + | + |  |  |  |  |  | 8 | 6 |
| *Urtica pilulifera* | Golan heights |  | + |  |  |  |  |  | + | 5 | 9 |
| *Rosa canina* | Golan heights | + | + | + | + |  |  |  | + | 0 | 8 |
| *Malva nicaeensis* | Golan heights | + | + | + | + |  |  |  | + | 15 | 3 |
| *Calicotome villosa* | Commercial company |  |  |  |  |  | + |  |  | 1 | 0 |
| *Mandragora autumnalis* | Commercial company |  |  |  |  |  | + |  |  | 5 | 0 |
| *Styrax officinalis* | Commercial company |  |  |  |  |  | + |  |  | 1 | 2 |
| *Ziziphus lotus* | Commercial company |  |  |  |  |  | + |  |  | 2 | 0 |
|  | | | | | | | | | Total | 146 | 156 |
|  | | | | | | | | | | 302 | |
